# Gender differences in adiponectin levels among obese individuals and their association with cardiovascular disease

**DOI:** 10.3389/fcell.2026.1923572

**Published:** 2026-08-27

**Authors:** Yujie Sun, Haoming Yu, Wenyao Sui, Jihong Li, Haoxuan Sui, Shengyu Han, Lei Wan, Yuhua Chi

**Affiliations:** 1 Department of General Medicine, Affiliated Hospital of Shandong Second Medical University, Weifang, Shandong, China; 2 School of Clinical Medicine, Shandong Second Medical University, Weifang, Shandong, China

**Keywords:** adiponectin, adiponectin paradox, cardiovascular disease, obesity, sexual dimorphism

## Abstract

Obesity is one of the major risk factors for cardiovascular disease Adiponectin, an adipokine with cardioprotective effects, exhibits significant sex differences, with higher plasma levels observed in women. However, the mechanistic basis of this sex difference and its pathophysiological significance in obesity-related cardiovascular disease require further elucidation. This review systematically examines the gender-specific pathways ranging from gonadotropins to adiponectin expression and signalling, as well as how dysregulation of these pathways in the context of obesity leads to cardiac damage. We propose that dysfunction of epicardial adipose tissue acts as a key hub, with local inflammation and alterations in adipokine signalling exacerbating coronary atherosclerosis, myocardial fibrosis and arrhythmias. Furthermore, we have examined the clinical conundrum known as the adiponectin paradox in advanced cardiometabolic diseases. In summary, a deeper understanding of the gender dimorphism of adiponectin is crucial for recognising gender-specific susceptibilities and may provide a strategic basis for future approaches to addressing obesity-mediated cardiovascular complications.

## 1 Introduction

Obesity, a multifactorial, complex and chronic metabolic disease, is showing an upward trend in prevalence worldwide. According to data from the World Health Organisation, approximately 2.548 billion adults worldwide were overweight or obese (BMI ≥25 kg/m^2^), and this figure is projected to reach 2.9 billion by 2030. The prevalence of obesity varies by gender, with adult women generally having a higher prevalence than men across all age groups (World Obesity Federation, 2025). Excessive consumption of high-calorie foods, a lack of physical activity, and significant changes in global food production practices have led to rising obesity rates (Wang et al., 2019). Adiponectin is a protein secreted primarily by fat cells in white adipose tissue (Guerre-Millo, 2002). Circulating adiponectin is a key marker of adipose tissue health and metabolic flexibility. As the principal beneficial hormone secreted by adipose tissue, genetically determined high levels of adiponectin can counteract the adverse effects of obesity (Straub and Scherer, 2019). Although previous literature has described gender differences in obesity and in adiponectin levels among obese individuals (Bjune et al., 2022; Ter Horst et al., 2020), analysis of the specific mechanisms underlying these gender differences remains insufficient. Although cardiovascular complications are one of the most clinically significant consequences of obesity, obesity is increasingly recognised as a systemic metabolic disease involving multiple organs (Sarma et al., 2021). Excessive fat leads to metabolic disturbances in various tissues; adipose tissue dysfunction, hepatic steatosis and alterations in the gut microbiota are the primary manifestations of obesity-related metabolic diseases (Eslami et al., 2026). Through mechanisms involving chronic inflammation, ectopic lipid deposition and alterations in endocrine signalling, these abnormalities lead to systemic metabolic imbalances and cardiovascular metabolic complications. As an endocrine factor derived from adipose tissue, adiponectin may act as a key mediator linking adipose tissue dysfunction to systemic metabolic imbalances and cardiovascular remodelling. This article will systematically elucidate, from a microscopic perspective, the disease pathway from sex hormones to adiponectin and its impact on obesity or metabolism, ultimately leading to cardiovascular disease, and explore the clinical application potential of adiponectin.

## 2 Adiponectin and its associated pathways and physiological functions

**FIGURE 1:**
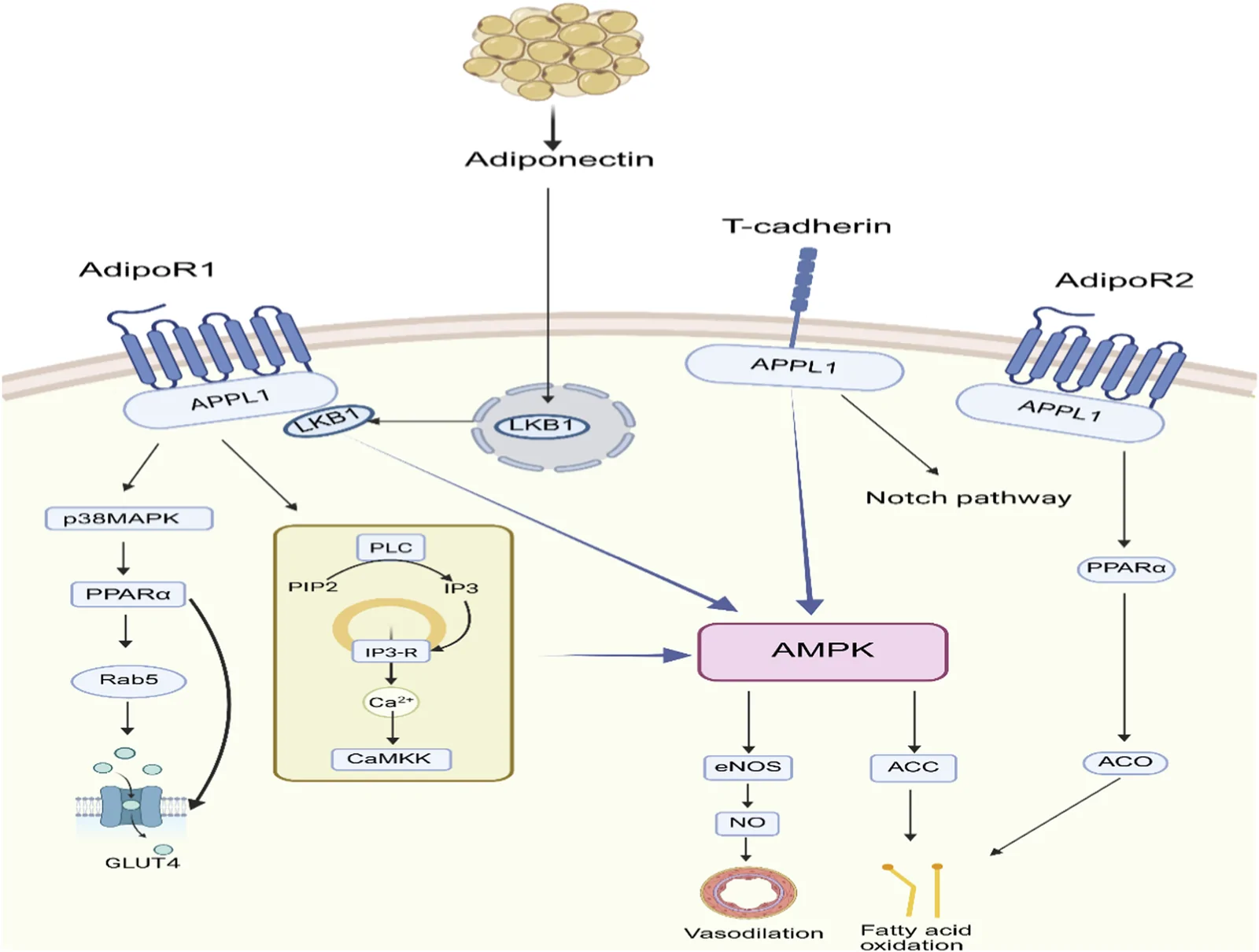
Adiponectin associated signaling pathways. Adiponectin acts on three receptors: AdipoR1, AdipoR2, and T-cadherin. APPL1 is a ligation protein that assists in mediating the adiponectin pathway. AdipoR1 can further phosphorylate p38MAPK by binding to APPL1 and further activate PPARα. AMPK can also be activated via LKB and PLC; AdipoR2 can bind to APPL1, activate PPARα, and further activate Acyl-CoA oxidase (ACO), an enzyme that catalyzes the first step of fatty acid peroxisome β-oxidation and makes the rate determine the step. T-cadherin can bind to APPL1 to activate the AMPK and Notch pathways. AdipoR1/2, adiponectin receptor 1/2; APPL1, adaptor protein containing PH domain, PTB domain and leucine zipper motif 1; AMPK, AMP-activated protein kinase; LKB1, liver kinase B1; eNOS, endothelial nitric oxide synthase; MAPK, Mitogen-activated protein kinase; NO, nitric oxide; ACC, acetyl-CoA carboxylase; PPARα, peroxisome proliferator-activated receptor alpha; ACO, acyl-CoA oxidase; PI3K, phosphoinositide 3-kinase; Akt, protein kinase B; PLC, phospholipase C; IP3, inositol 1,4,5-trisphosphate; CaMKK, Ca2+/calmodulin-dependent protein kinase; GLUT4, glucose transporter type 4.

Adiponectin circulates in the blood in the form of full-length trimers, hexamers, high-molecular-weight multimers and spherical fragments. High-molecular-weight multimers are the primary bioactive forms responsible for its insulin-sensitising and cardioprotective effects (Nguyen, 2020), whilst overexpression of spherical adiponectin can significantly reduce the formation of atherosclerotic lesions (Zhao et al., 2015). Adiponectin possesses a wide range of physiological functions, primarily including the promotion of fatty acid biosynthesis, the inhibition of hepatic gluconeogenesis, and anti-atherosclerotic, anti-apoptotic, anti-fibrotic and anti-inflammatory effects (Funcke and Scherer, 2019; Santoro et al., 2019). Adiponectin receptors include adiponectin receptors (AdipoR) 1, AdipoR2 and T-cadherin. Phosphorylated tyrosine-PH domain and leucine zipper-interacting protein 1 (APPL1) can bind to adiponectin receptors and positively mediates adiponectin signal transduction in mammals (Ramakrishnan et al., 2026). The downstream signalling pathways mediated by adiponectin primarily include the AMP-activated protein kinase (AMPK) pathway, the peroxisome proliferator-activated receptor α (PPARα) pathway, the phosphatidylinositol 3-kinase (PI3K)–protein kinase B (Akt) pathway mediated by the APPL adaptor protein, and the mitogen-activated protein kinase (MAPK) pathway. See Figure 1.

## 3 Gender differences in fat tissue distribution

Depending on their location, adipose tissues can be classified as subcutaneous adipose tissue and visceral adipose tissue. Compared with women of the same total body fat mass, men may have higher levels of visceral fat (Mauvais-Jarvis et al., 2013). This phenomenon is primarily associated with elevated androgen levels in men; androgens promote the accumulation of adipose tissue in the visceral region and reduce the secretion of adiponectin by fat cells (Chávez-Guevara et al., 2023). Women have more subcutaneous fat than men; this characteristic confers an evolutionary advantage, helping to stabilise energy reserves to support reproduction and lactation. Before the menopause, women have higher insulin sensitivity and lower fasting blood glucose levels than men; however, the menopause triggers changes in fat distribution, elevated blood pressure, increased blood lipid levels and impaired glucose tolerance (Bauzá-Thorbrügge et al., 2024). After the menopause, women’s fat distribution shifts towards a phenotype more similar to that of men, whilst oestrogen replacement therapy can prevent the accumulation of intra-abdominal fat.

Lipoprotein lipase (LPL), as the rate-limiting enzyme for fat storage, exhibits sex-specific activity: in women, subcutaneous LPL activity is higher than abdominal LPL activity; whereas in men, testosterone inhibits subcutaneous LPL, thereby promoting abdominal LPL activity. This regulatory pattern also applies to fat mobilisation. Oestradiol upregulates anti-lipolytic α2-adrenergic receptors in female subcutaneous fat, thereby establishing a higher ratio of anti-lipolytic to pro-lipolytic receptors; consequently, catecholamine-stimulated lipolysis is attenuated compared with that in men. These differences enable subcutaneous fat in the lower limbs of premenopausal women to act as a stable metabolic reservoir, exhibiting greater expansion capacity during energy surplus but a weaker lipolytic response (Palmer and Clegg, 2015). Lower-limb adiposity may act as a metabolic buffer for dietary fat or lipids, protecting other tissues from lipotoxic damage caused by ectopic fat deposition and lipid spillover. The central oestrogen receptor α (ERα) also plays a significant role in determining fat distribution. Studies have further shown that oestradiol can regulate thermogenic adipose tissue at the central level by activating AMPK in the hypothalamus. ERα expression is more closely associated with neurons in visceral adipose tissue, and studies have shown that oestrogen promotes lipolysis by increasing sympathetic nerve activity in adipose tissue. Male rats have more neural projections to visceral fat, whilst female rats have more neural projections to subcutaneous fat stores (Neeland et al., 2018). Furthermore, whilst ERα mediates lipolysis, ERβ exerts the opposite effect by inhibiting the peroxisome proliferator-activated receptor γ (PPARγ), thereby further reducing the expression of the adiponectin gene; however, there is currently limited research into the regulation of adiponectin gene expression by ERα and testosterone.

## 4 Classification of adipose tissue and gender differences

Adipose tissue can be classified into three types according to function: white, beige and brown. The browning or beige-ing of white adipose tissue (WAT) refers to its acquisition of characteristics similar to those of brown adipose tissue (BAT), including an increase in the number of mitochondria and upregulation of uncoupling protein 1 (UCP1); beige adipocytes are a hybrid type of adipocyte typically found within white adipose tissue reservoirs. Enhancing the activation of brown adipose tissue and promoting the browning of white adipose tissue can influence energy homeostasis and help prevent obesity and obesity-related metabolic diseases (Singh et al., 2021). However, obesity impairs the structure and function of brown adipose tissue, causing it to whiten and reducing its thermogenic capacity (Kononova et al., 2024). White adipose tissue possesses a basal rate of intracellular lipolysis, which increases with adipocyte hypertrophy, a phenomenon particularly pronounced in obese men, thereby leading to elevated plasma free fatty acid levels. In addition to this basal rate, sympathetic activation and hormonal signalling further stimulate lipolysis. The lipolytic response to catecholamines varies depending on the location of the adipose tissue, being more pronounced in visceral white adipose tissue than in subcutaneous white adipose tissue. In a state of obesity, increased β-adrenergic receptor sensitivity amplifies this response, particularly in visceral adipocytes (v et al., 2017).

During the proliferation of white adipose tissue caused by obesity, the differentiation of macrophages (MPs) shifts from the anti-inflammatory M2 phenotype to the pro-inflammatory M1 phenotype. Testosterone further promotes M1 differentiation by regulating G proteins and inhibits preadipocyte differentiation via Akt phosphorylation (Ren et al., 2017). Owing to differences at the hormonal and cellular levels, women typically have higher brown adipose tissue (BAT) content and greater non-shivering thermogenic activity than men. Sex hormones regulate brown adipose tissue in a sex-specific manner: oestrogen promotes browning, whilst androgens inhibit it. Oestrogen enhances browning via hypothalamic pathways, including brain-derived neurotrophic factor (BDNF)-mediated stimulation of cardiac natriuretic peptide (NP), thereby upregulating mitochondrial biogenesis. Consequently, women exhibit higher brown adipose tissue activity, although this difference diminishes with age (Koceva et al., 2024). Compared with men, women have a greater number of inducible brown adipocytes in their subcutaneous fat, as well as higher levels of mitochondrial and thermogenic gene expression. They are also more sensitive to cold, and oestradiol levels are positively correlated with cold-induced thermogenesis, thereby promoting the browning of fat cells (Herz et al., 2021). Although the primary functions of brown adipose tissue (BAT) are thermogenesis and metabolic regulation, the mass of BAT in adults is limited and varies between individuals. Although cold stimulation can moderately promote the browning of white adipose tissue, the role of brown adipose tissue in metabolic homeostasis is not limited to the cold stress response; it remains under the control of the sympathetic nervous system, and its activity declines with age (Scheele et al., 2020). This renders cold-stimulation-based strategies less suitable for the elderly or those at high cardiovascular risk, and increasing brown adipose tissue content does not significantly reverse obesity.

## 5 Gender differences in the pathophysiological changes of adipocytes

In obese individuals, male adipocytes are characterised by hypertrophy and are mainly distributed in the abdomen; in contrast, female adipocytes are primarily characterised by hyperplasia and are mainly distributed in the buttocks and thighs (Gavin and Bessesen, 2020). White adipocytes adapt to metabolic demands through hypertrophy and atrophy, and their size directly reflects their functional state (Gliniak et al., 2023). When adipocytes are unable to proliferate and safely store lipids, obesity-related complications arise, leading to the abnormal accumulation of lipids in other tissues and thereby causing lipotoxicity. Adipocyte hypertrophy induces hypoxia, thereby triggering the release of factors such as tumour necrosis factor-alpha (TNF-alpha), interleukin-6 (IL-6) and vascular endothelial growth factor (VEGF) from adipose tissue to stimulate angiogenesis and extracellular matrix (ECM) remodelling, thereby supporting tissue expansion. Initially, this response mediated by hypoxia-inducible factor 1alpha (HIF-1alpha) and including the upregulation of matrix metalloproteinase-14 (MMP-14) activity is adaptive. However, in cases of persistent or severe obesity, hypoxia can no longer be compensated. During this phase of maladaptation, vascular endothelial growth factor (VEGF) promotes vascular dysfunction, thereby providing a microenvironment for immune cells; at the same time, endothelial cells may undergo mesenchymal transition, and these factors collectively exacerbate inflammation and fibrosis. A key mediator in advanced obesity is endotrophin, which is produced when matrix metalloproteinase 14 (MMP14) cleaves type VI collagen (COL6) in the extracellular matrix (ECM). Endotrophin strongly drives local fibrosis and inflammation, processes that are closely associated with increased cardiovascular and all-cause mortality. Concurrently, excessive extracellular matrix deposition displaces perilipin-1 from around the lipid droplets, whilst increased mechanical stress activates caspase-6, thereby inducing adipocyte apoptosis. Subsequently, recruited M1-type macrophages clear the dead fat cells, thereby maintaining a state of chronic, low-grade inflammation (Sun et al., 2023).

Inflammation promotes extracellular matrix (ECM) deposition and fibrosis. Factors such as IL-6 can induce mesenchymal transformation in fibroblasts, whilst transforming growth factor β (TGF-β) directly promotes excessive ECM production. Obesity enhances the secretion of TGF-β by stromal cells within adipose tissue and upregulates TGF-β expression in adipocytes. Immune cells that infiltrate adipose tissue during obesity often persist even after weight loss, thereby sustaining inflammation and hindering the resolution of fibrosis. Fibrosis of adipocytes is largely irreversible; it severely limits the tissue’s distensibility and lipid storage capacity, promotes ectopic lipid deposition in other organs, and leads to systemic metabolic diseases. Adiponectin exerts dual anti-inflammatory and anti-fibrotic effects. Spherical adiponectin primarily exerts its signalling effects via AdipoR1, reducing the expression of Toll-like receptor 4 (TLR4) in macrophages and inhibiting inflammation via an IL-1-haem oxygenase-1 (HO-1)-dependent pathway. Full-length adiponectin, on the other hand, acts primarily via AdipoR2, driving the polarisation of macrophages towards the M2 phenotype via the IL-4-signal transduction and STAT6 signalling pathways (Mandal et al., 2011).

**FIGURE 2:**
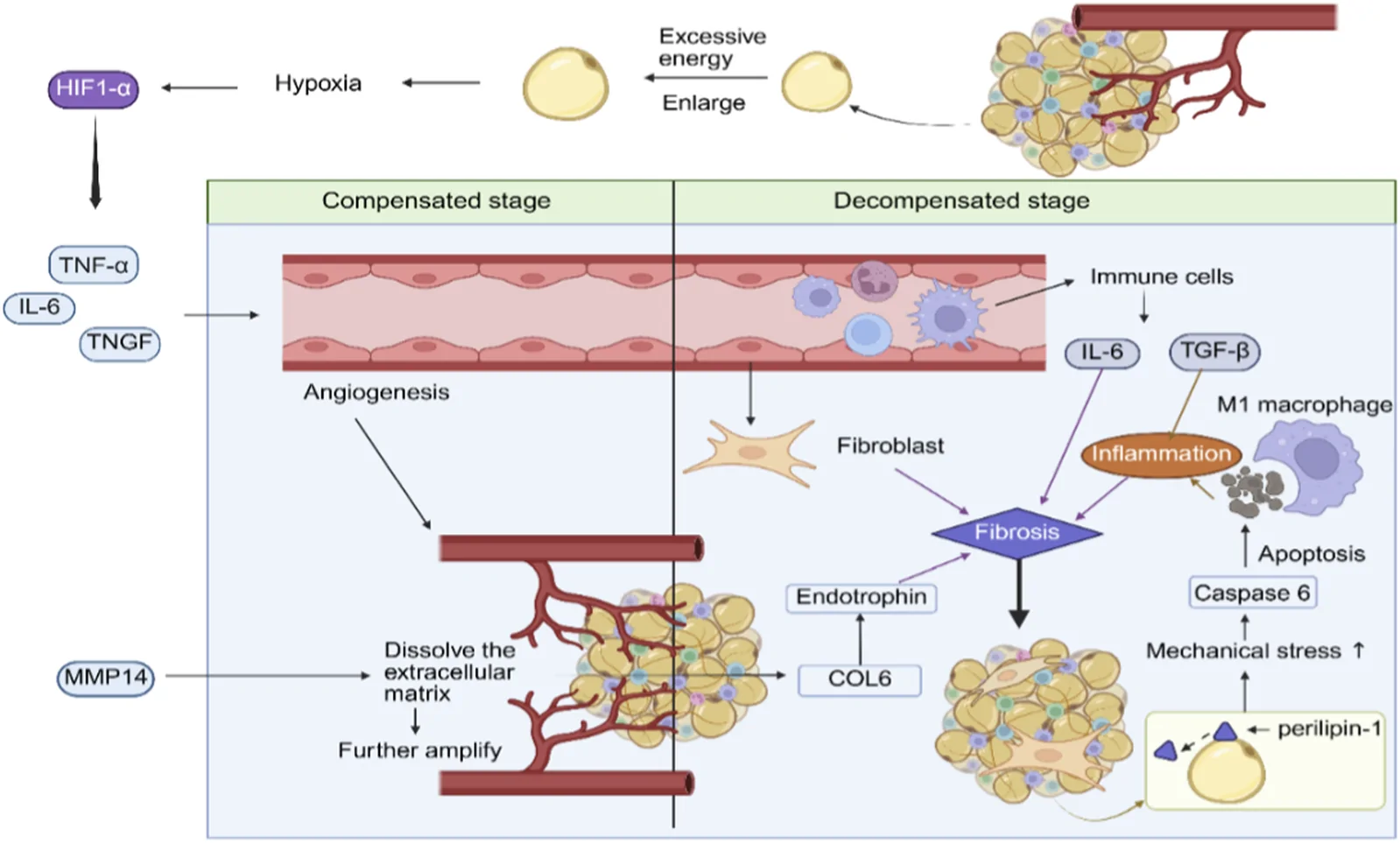
Gender differences the pathophysiological changes of adipocytes. This schematic illustrates the proposed progression: (1) Compensated cellular hypertrophy leads to relative hypoxia and HIF activation. (2) This initiates angiogenic and profibrotic responses (e.g., VEGF, COL6). (3) Ultimately, these processes exceed compensatory capacity, resulting in extracellular matrix dissolution, aberrant fibrotic remodeling, and sustained inflammation. Arrows indicate proposed stimulatory relationships. Abbreviations: COL6, Collagen VI; HIF-1alpha, Hypoxia-Inducible Factor-1Alpha; M1, Macrophage Type 1; IL-6, Interleukin-6; MMP14, Matrix Metalloproteinase-14; TGF-beta, Transforming Growth Factor-Beta; TNF-alpha, Tumor Necrosis Factor-Alpha.

Proliferative adipocytes are smaller in size, more sensitive to insulin, and possess greater storage capacity. The abundance of these cells in subcutaneous fat indicates good tissue expandability and is associated with a higher capillary density. Conversely, HIF stability is negatively correlated with a healthy adipokine profile, macrophage recruitment and insulin sensitivity, whilst oestrogen can attenuate this relationship (Palmer and Clegg, 2015). In summary, women have a higher proportion of subcutaneous fat, which provides greater safety in terms of fatty acid storage and is less likely to lead to obesity-related complications. In contrast, men tend to accumulate more visceral fat, making them more susceptible to obesity-related complications. See Figure 2.

## 6 Gender differences in adiponectin expression in obese individuals

Different genders exhibit distinct fat storage patterns, leading to differences in fat distribution. In obese individuals, adiponectin levels are reduced in both men and women; however, women’s adiponectin levels remain higher than those of men. In men, levels of pro-inflammatory substances such as IL-6 are significantly elevated (Ter Horst et al., 2020). There is a significant difference in the rate of adiponectin secretion between abdominal fat cells and subcutaneous fat cells (Bellissimo et al., 2021), indicating impaired adiponectin signalling. Compared with men, women have higher levels of insulin receptor, Akt2 and glucose transporter type 4 (GLUT4) in their subcutaneous adipose tissue, thereby enhancing glucose uptake capacity. Furthermore, oestrogen promotes MAPK phosphorylation, thereby increasing GLUT4 expression and, consequently, enhancing glucose uptake signalling (Nicolaisen et al., 2024). These overlapping signalling pathways (PI3K/Akt and MAPK) suggest that oestrogen and adiponectin may exhibit convergent or synergistic effects in regulating glucose metabolism. Whether oestrogen exerts its effects by raising adiponectin levels, by binding to oestrogen receptors on fat cells, or through synergistic interaction with adiponectin remains to be confirmed by further research. Normal mitochondrial function is crucial for lipid metabolism, differentiation and adipokine secretion in fat cells. Overnutrition can overload the mitochondria, triggering the accumulation of reactive oxygen species (ROS), which in turn leads to macrophage infiltration and chronic inflammation. In contrast, female fat cells exhibit superior mitochondrial parameters, including enhanced thermogenic gene expression, greater functional volume and stronger antioxidant capacity. The higher mitochondrial activity in women compared to men mitigates oxidative tissue damage.

During the menopausal transition in women, ovarian function gradually declines, leading to reduced secretion of sex hormones and the loss of the inhibitory effect of follicle-stimulating hormone (FSH). At the same time, a decrease in inhibin B levels further weakens the inhibitory effect on FSH. Prior to menopause, oestradiol (E2) and FSH levels in obese women are typically lower than in non-obese women. After the menopause, adipose tissue converts androgens into oestrogens via peripheral aromatisation; at the same time, obesity also inhibits the synthesis of sex hormone-binding globulin, thereby accelerating the clearance of E2. This promotes the accumulation of cholesterol and reduces the ratio of total cholesterol to high-density lipoprotein. As E2 levels decline, the expression of genes associated with β-oxidation is downregulated, leading to insufficient oxidation of free fatty acids released from visceral fat. As fat mass increases, lean body mass in the whole body and the trunk falls below pre-menopausal levels. Furthermore, reduced circulating adiponectin levels may be associated with more severe menopausal symptoms (Ko and Jung, 2021; Palacios et al., 2024; Opoku et al., 2023).

**FIGURE 3:**
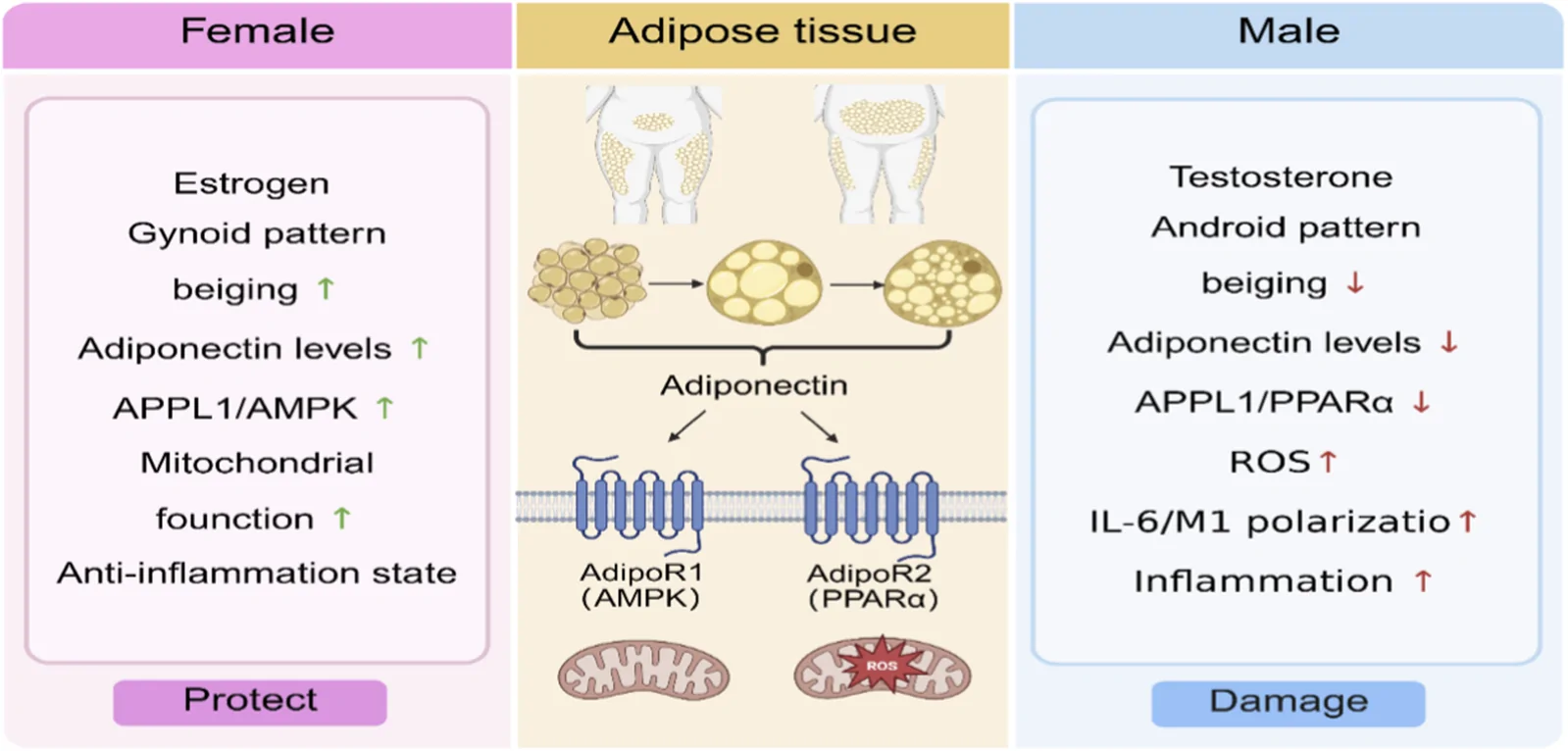
Hormonal changes in people with obesity adiponectin. This schematic compares sex-dependent differences in adipose tissue distribution and function in obesity. In females, estrogen promotes a gynoid fat distribution pattern characterized by enhanced adipose tissue browning, increased adiponectin secretion, and activation of the APPL1-AMPK signaling axis. These changes are associated with improved mitochondrial function, enhanced oxidative capacity, and an overall anti-inflammatory metabolic state. In contrast, male obesity is characterized by an android fat distribution pattern, reduced adipose browning capacity, decreased adiponectin levels, and impaired APPL1-PPARα signaling. These alterations are associated with increased reactive oxygen species production, macrophage M1 polarization driven by IL-6, and a chronic pro-inflammatory state. Together, these sex-specific differences provide a mechanistic framework for the generally lower cardiometabolic risk observed in women with obesity compared with men. Abbreviations: ROS, reactive oxygen species.

Although the effects of ageing on the male reproductive system manifest later in life, testosterone levels still decline with age. This decline promotes the accumulation of abdominal fat and is negatively correlated with obesity. The combined decline in testosterone and adiponectin levels reduces mitochondrial function, thereby leading to metabolic syndrome and an increased risk of cardiovascular disease (Kuribayashi et al., 2024). For symptomatic obese postmenopausal individuals, combining adiponectin replacement therapy with hormone replacement therapy may improve clinical outcomes. At the same time, the specific epigenetic and post-transcriptional mechanisms through which sex hormones regulate the expression and secretion of adiponectin remain to be further elucidated. See Figure 3.

## 7 Adiponectin in cardiac remodelling and the progression of heart failure

**FIGURE 4:**
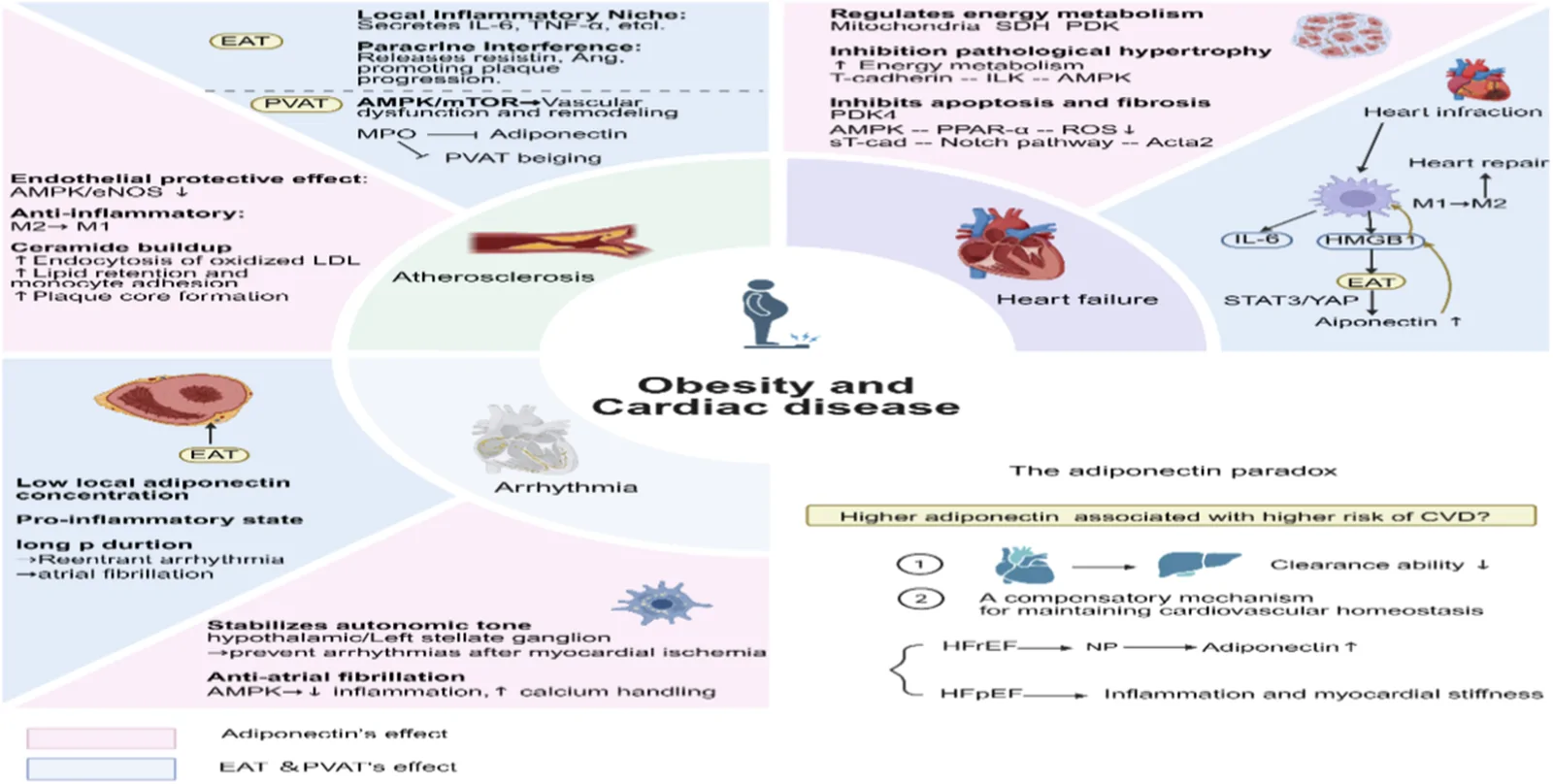
Adiponectin in cardiac remodelling and the progression of heart failure. The figure contrasts the pathogenic mechanisms driven by dysfunctional cardiac-associated adipose tissue against the cardiometabolic benefits mediated by adiponectin signaling. It further contextualizes the paradoxical association of high adiponectin levels with poor prognosis in advanced cardiovascular disease. The pink area primarily represents the effects of adiponectin, while the blue area mainly indicates the roles of EAT and PAVT. Abbreviations: Acta2, Actin alpha 2, smooth muscle; Ang, Angiotensi; CVD, Cardiovascular disease; EAT, Epicardial adipose tissue; HFpEF, Heart failure with preserved ejection fraction; HFrEF, Heart failure with reduced ejection fraction ILK, Integrin-linked kinase; M1/M2, Macrophage type 1/2; MPO, Myeloperoxidase; mTOR, Mammalian target of rapamycin; NP, Natriuretic peptide; PDK4, Pyruvate dehydrogenase kinase 4; PVAT, Perivascular adipose tissue; SDH, Succinate dehydrogenase; sT-cad, Soluble T-cadherin; STAT3, Signal transducer and activator of transcription 3; YAP, Yes-associated protein.

Obesity is a major risk factor for cardiovascular disease (CVD), the onset of which is primarily driven by atherosclerosis, myocardial metabolic disorders and vascular dysfunction. As a key protective adipokine, reduced levels of adiponectin establish a crucial link between obesity and cardiovascular disease. We will explore the potential gender differences in the cardioprotective effects of adiponectin which counteract atherosclerosis, improve myocardial metabolism and maintain the function of perivascular adipose tissue with a view to guiding targeted interventions. See Figure 4.

### 7.1 Adiponectin and atherosclerosis

Obesity promotes atherosclerosis through multiple mechanisms, including inflammatory activation, lipotoxicity and dysbiosis of the gut microbiota. Obesity-induced chronic low-grade inflammation leads to elevated levels of pro-inflammatory cytokines, such as TNF-alpha and IL-6, which promote endothelial dysfunction, oxidative stress and inflammation of the vascular wall. Obesity-associated insulin resistance further exacerbates dyslipidaemia and vascular dysfunction. Furthermore, an imbalance in the gut microbiota may also promote the circulation of microbial metabolites such as lipopolysaccharides and trimethylamine N-oxide (TMAO) by compromising the intestinal barrier. Among these, TMAO promotes cholesterol accumulation in macrophages, foam cell formation, and enhances prothrombotic responses, thereby further accelerating the formation of atherosclerotic plaques. These processes are accompanied by adipose tissue dysfunction and reduced levels of adiponectin, thereby undermining its anti-inflammatory, antioxidant and endothelial protective effects. In addition to obesity-related metabolic abnormalities, the incidence of coronary artery disease (CAD) also exhibits significant gender differences. Epidemiological studies indicate that premenopausal men typically have a higher risk of CAD than women, whereas postmenopausal women have a significantly higher risk, suggesting that sex hormones play an important role in vascular protection. The decline in oestrogen levels following menopause may promote endothelial dysfunction, inflammatory activation and dysregulation of lipid metabolism; by affecting the endocrine function of adipose tissue, it further impairs adiponectin-mediated vascular protection and accelerates the progression of atherosclerosis, thereby reducing adiponectin levels (Naderian et al., 2025).

Obesity disrupts the interaction between endothelial cells and adipocytes by altering the secretion and function of extracellular vesicles (EVs), thereby leading to adipocyte dysfunction. Exosomes derived from endothelial cells typically maintain the health of adipocytes by regulating inflammation, angiogenesis and metabolic balance. In a state of obesity, these protective mechanisms are compromised and the release of endothelial cell exosomes is impeded; this process may be influenced by adiponectin (Naidu et al., 2025). Adiponectin exerts its cardioprotective effects primarily through two key mechanisms. Firstly, by activating AMPK via APPL1, it enhances the activity of endothelial nitric oxide synthase (eNOS) and promotes the production of nitric oxide (NO), thereby facilitating vasodilation and maintaining endothelial function. At the same time, the balance between NO and ROS must not be overlooked. Physiological levels of NO promote coronary vasodilation, enhance oxygen supply to the myocardium, and improve mitochondrial efficiency. When NO levels are low, it reduces coronary blood flow and increases vascular tone, both of which are key features of the progression of heart failure. At low levels, ROS can promote cell survival pathways, but elevated levels damage endothelial cells and reduce the availability of eNOS, leading to the production of peroxynitrite, which impedes vasodilation; persistent ROS activation leads to fibroblast activation, extracellular matrix deposition and ventricular remodelling. Adiponectin deficiency can increase ROS production and promote NF-κB expression, further activating the NOD-like receptor thermal protein domain-associated protein 3 inflammasome, releasing IL-1β and IL-18, and inducing pyroptosis (Naderian et al., 2026).

Secondly, adiponectin binds to T-cadherin, which is GPI-anchored to the vascular wall, thereby mediating its localisation and counteracting damage caused by hypercholesterolaemia (Kondo et al., 2025); this pathway inhibits the formation of foam cells by reducing the accumulation of lipid droplets and the cholesterol load within macrophages (Liu et al., 2022). When adiponectin levels are reduced, T-cadherin binds to low-density lipoprotein (LDL) and promotes atherosclerosis (Cullen et al., 2023a). Spherical adiponectin primarily binds to T-cadherin; overexpression of spherical adiponectin increases energy expenditure and food intake, whilst significantly alleviating atherosclerotic lesions (Yamauchi et al., 2003).

Hyperlipidaemia increases endothelial permeability, promoting the migration of monocytes and their differentiation into macrophages. These macrophages phagocytose oxidised low-density lipoprotein (oxLDL), thereby transforming into foam cells, releasing inflammatory factors and exacerbating local inflammation. In adipose tissue, the polarisation of macrophages varies according to metabolic status. Under normal conditions, anti-inflammatory M2 adipose tissue macrophages (ATMs) predominate, whereas obesity increases the number of pro-inflammatory M1 ATMs and reduces M2 ATMs, thereby shifting the balance towards inflammation (Feng et al., 2020). M2 macrophages secrete anti-inflammatory factors such as IL-10 to counteract the inflammation driven by M1 macrophages. The Tribbles pseudo-kinase 1 gene promotes adiponectin synthesis through post-transcriptional regulation (Hernandez-Resendiz and Burkhardt, 2024), and its expression is associated with tissue-resident M2-like macrophages, which contribute to endothelial repair and plaque remodelling (Prete et al., 2025). The response of macrophages to adiponectin is gender-specific: adiponectin-treated male macrophages exhibit attenuated AdipoR2-PPARα signalling and heterogeneous inflammatory characteristics, whereas female macrophages upregulate AdipoR1 without altering AdipoR2-PPARα (Gianopoulos and Daskalopoulou, 2025), a finding associated with cardiovascular disease in men. Ceramide levels are elevated in the adipose tissue of obese patients. Ceramides inhibit insulin signalling and promote the uptake of oxidised low-density lipoprotein (oxLDL), thereby facilitating lipid retention and monocyte adhesion—key steps in plaque formation. Specific types of ceramides can predict the severity of cardiovascular disease; therefore, inhibiting ceramide production may help to improve atherosclerosis. Ceramidase shares sequence homology with the adiponectin receptor, suggesting that adiponectin may alleviate atherosclerosis by activating ceramidase to degrade ceramides (Chaurasia and Summers, 2021).

Obesity leads to ectopic fat deposition, including thickening of epicardial adipose tissue (EAT), a layer of fat situated between the myocardium and the visceral pericardium, which shares its blood supply with the myocardium (Liu et al., 2025). The accumulation of EAT is associated with impaired myocardial blood flow reserve, increased coronary plaque vulnerability, coronary artery calcification, and an increased overall severity of coronary artery disease (Gao and Fang, 2025). At the same time, obesity also leads to structural and functional changes in perivascular adipose tissue (PVAT), resulting in the loss or attenuation of its anti-myocardial contractile properties (Almabrouk et al., 2018). Both of these conditions are characterised by reduced levels of adiponectin in obese patients.

Studies have shown that EAT volume is positively correlated with the presence of high-risk plaques (Konwerski et al., 2022). EAT primarily acts by secreting pro-inflammatory and pro-atherosclerotic factors such as angiotensinogen, IL-6, TNF-alpha and lipoproteins, and by acting as a source of inflammatory responses mediated by nuclear factor kappaB (NF-kappaB) and c-Jun N-terminal kinase (JNK), which activate B cells. The progression of atherosclerosis involves a phenotypic transition from brown adipose tissue to white adipose tissue. IL-6 drives this transition by activating the Janus kinase (JAK)/STAT3 pathway, thereby promoting the development of focal atherosclerosis. NF-kappaB further stimulates the expression of IL-8, which recruits monocytes and induces their firm adhesion to endothelial cells,a crucial early step in plaque formation (Yan et al., 2024). Adiponectin enhances vascular function by activating AMPK and phosphorylating eNOS, thereby inducing hyperpolarisation of smooth muscle cells and improving endothelial cell activity. Under normal conditions, AMPK inhibits the activity of inflammatory cytokines such as TNF-alpha and IL-6, which are key factors in regulating adiponectin levels. A high-fat diet reduces AMPK activity in PVAT, potentially promoting vascular dysfunction and remodelling via the AMPK/mTOR pathway. Consequently, PVAT can promote vascular homeostasis only when adipokine balance is maintained; once this balance is disrupted, it exerts adverse effects on the vascular system (Sowka and Dobrzyn, 2021). In patients with coronary heart disease, PVAT exhibits high levels of adiponectin expression despite low serum adiponectin levels. Low expression of adipokine receptors triggers compensatory upregulation; in disease states, impaired function of molecular chaperones delays the release of adipokines from the endoplasmic reticulum (Gruzdeva et al., 2022). Furthermore, PVAT exhibits a phenotype similar to that of brown adipose tissue (BAT), which, upon exposure to cold, increases energy expenditure and releases vasoactive substances such as prostacyclin, thereby mitigating atherosclerosis (Chang et al., 2012).

Myeloperoxidase (MPO) is a key promoter of atherosclerosis and an important risk predictor for acute coronary syndrome. In obese individuals, reduced adiponectin secretion promotes the release of MPO from perivascular adipose tissue (PVAT), which in turn further inhibits adiponectin production via a negative feedback mechanism, thereby exacerbating endothelial dysfunction. MPO inhibits the browning of PVAT in obese mice by oxidising soluble guanylate cyclase and reducing the expression of its β1 subunit (Hof et al., 2025). MPO inhibitors may therefore alleviate obesity-related endothelial dysfunction and delay atherosclerosis. MPO levels are significantly elevated in obese men, whereas this phenomenon is not observed in women (Hertiš et al., 2024), this suggests the existence of a gender-specific mechanism, which should provide a basis for targeted interventions. Dipeptidyl peptidase-4 (DPP4) is highly expressed in EAT/PVAT, making it a promising therapeutic target. DPP4 inhibition may influence cardiovascular disease (CVD) by inducing glucagon-like peptide-1 (GLP-1) to upregulate adiponectin, promote M2 macrophage polarisation, and reduce lipid accumulation. Experimental studies have further demonstrated that the removal of EAT significantly reduces myocardial levels of IL-1β and IL-6, supporting the key role of EAT in amplifying local cardiac inflammation (Zhao et al., 2024). Exogenous adiponectin also reduces levels of TNF-alpha and IL-6, thereby alleviating cardiac inflammation (Yan et al., 2025). It is worth noting that preclinical studies suggest that GLP-1R/GLP-2R dual agonists are superior to GLP-1R agonists alone in reducing EAT volume and inflammatory cell infiltration, indicating that simultaneous activation of these two incretin pathways may exert a more effective regulatory effect on EAT inflammation. Furthermore, obesity itself can lead to reduced GLP-1 secretion and decreased signalling sensitivity. With regard to gender differences, evidence suggests a broad and significant synergistic effect between oestrogen and the GLP-1 system: GLP-1-based incretin therapy is often more effective in women than in men. This indirectly suggests that oestrogen may enhance the body’s responsiveness to GLP-1 signalling or promote its downstream metabolic effects, although the specific molecular mechanisms remain to be elucidated (Goulet et al., 2026). In summary, these findings indicate that the DPP4/GLP-1–EAT axis may influence the progression of cardiovascular disease by regulating adipose tissue inflammation and macrophage polarisation.

Men generally exhibit greater visceral adipose tissue (VAT) and epicardial adipose tissue (EAT) than women, but the association between these fat storage sites and systemic inflammation is weaker in men. In contrast, the more visceral fat women have, the higher their levels of inflammation; this difference may be explained by the protective effects of oestrogen. Before the menopause, oestrogen tends to promote the storage of subcutaneous fat rather than visceral fat, and helps to reduce inflammation; subcutaneous fat also produces oestrogen via aromatase. After the menopause, falling oestrogen levels lead to a masculinised fat distribution, characterised by an increase in visceral and abdominal subcutaneous fat, heightened systemic inflammation, and accelerated coronary artery calcification (Carbone et al., 2019). In obese individuals with cardiometabolic abnormalities, elevated IL-18 levels are associated with gut microbiota dysbiosis, characterised by a reduction in beneficial bacteria and an increase in pathogenic bacteria. Beneficial bacteria produce short-chain fatty acids and branched-chain amino acids, which help maintain metabolic health (Lara-Guzmán et al., 2025). Furthermore, elevated levels of lipopolysaccharide-binding protein (LBP) and the associated increase in IL-6 levels are directly linked to the risk of coronary atherosclerotic heart disease (Metz et al., 2025). Animal studies have shown that ageing leads to sex-specific changes in the transcriptional activity of genes associated with metabolism and inflammation in epicardial fat. In aged female rats, the expression of IL-1α, IL-1β and IL-6Rα in epicardial fat was significantly reduced, suggesting that the transcriptional profile in females exhibits lower pro-inflammatory characteristics (Kocher et al., 2017). Compared with women, male patients exhibited significantly reduced adiponectin gene expression in both epicardial adipose tissue (EAT) and peritoneal adipose tissue (PVAT). These results confirm, at the level of gene transcription, that men exhibit a more unfavourable adiponectin expression profile in the key epicardial adipose tissue pool.

Vascular smooth muscle cells (VSMCs) exhibit two phenotypes: one in a stable contracted state, and the other in a proliferative state, which secretes matrix metalloproteinases (MMPs) to degrade the extracellular matrix (ECM), thereby promoting migration towards the intima and exacerbating inflammation and atherosclerosis. Through paracrine action, adiponectin binds to AdipoR1/R2 and activates AMPK, thereby guiding VSMCs to differentiate towards a contractile, anti-atherosclerotic phenotype. Adiponectin deficiency triggers compensatory upregulation of AMPK; however, in the female aorta, this is accompanied by a marked upregulation of MMP3 and a significant downregulation of the extracellular matrix component desmin, suggesting that women with lower adiponectin levels may be more susceptible to atherosclerosis (Cullen et al., 2023b).

### 7.2 Adiponectin and heart failure

Under the sustained cardiac load caused by hypertension or obesity, the heart initially undergoes adaptive, compensatory myocardial hypertrophy to maintain cardiac output. However, this pathological hypertrophy triggers a vicious cycle leading to heart failure, cardiomyocytes begin to undergo apoptosis, accompanied by interstitial proliferation and fibrosis; the progression of these changes directly impairs myocardial contractility, causing cardiac function to shift from a compensated to a decompensated state, ultimately resulting in heart failure. For every one-unit increase in BMI, the risk of heart failure increases by 5% in men and 7% in women (Welsh et al., 2024), highlighting the importance of obesity as an independent risk factor for heart failure.

In a state of obesity, elevated levels of free fatty acids promote the onset of lipotoxicity. When excessive fatty acids enter non-adipose tissues, they can induce mitochondrial dysfunction, endoplasmic reticulum stress and inflammatory activation, whilst simultaneously reducing the secretion of adiponectin from adipose tissue, thereby further undermining its metabolic protective effects (Naderian et al., 2025). The cardioprotective effects of adiponectin also involve the regulation of autophagy. Mitochondrial autophagy degrades damaged mitochondria and increases the rate of mitochondrial turnover in cardiomyocytes, which is crucial for quality control (Tam et al., 2025). On the other hand, with regard to mitochondrial biosynthesis, the expression of many associated genes is transcriptionally regulated by PPARγ, co-activator-1α (PGC-1α) (Huang et al., 2024). The levels of activated PGC-1α and functional mitochondria are maintained by Sirtuin 1 (SIRT1) and AMPK, whilst elevated TNF-alpha levels are the primary cause of the obesity-induced decline in PGC-1α expression (Das et al., 2024). AMPK enhances mitochondrial respiration in cardiomyocytes via a succinate dehydrogenase (SDH/complex II)-dependent mechanism. SIRT3 promotes SDH assembly by upregulating the expression of succinate dehydrogenase assembly factor 1 (SDHAF1) via a SIRT3-dependent pathway, thereby enhancing energy production and improving the hypoxia tolerance of cardiomyocytes (Jeon et al., 2021). Fibroblast growth factor 21 (FGF21) is a single-transmembrane protein that acts primarily on adipose tissue. It exerts cardioprotective effects either directly or indirectly. It acts on cardiomyocytes via adiponectin and, by activating the PI3K/AKT signalling pathway, downregulates the production of pyruvate dehydrogenase kinase 4 (PDK4), which inhibits the pyruvate dehydrogenase complex. PDK4 deficiency improves left ventricular function and reduces remodelling following myocardial infarction; PDK4 silencing alleviates cardiac dysfunction and cardiomyocyte apoptosis, whilst its overexpression exacerbates calmodulin-dependent phosphatase-induced cardiac hypertrophy and fibrosis (Zhang K. et al., 2025).

Consequently, adiponectin coordinates mitochondrial homeostasis and mitigates damage by activating key regulatory pathways; it enhances mitochondrial biogenesis and renewal via the AMPK-SIRT1-PGC-1α pathway, stabilises SDH via the AMPK-SIRT3-SDHAF1-SDH axis, thereby improving the efficiency of the electron transport chain and promotes glucose oxidation by inhibiting PDK4 via the PI3K/AKT-PDK pathway. Overall, these adiponectin-mediated mechanisms collectively form a comprehensive network that is essential for mitochondrial quality control and metabolic adaptation in the heart.

### 7.3 Inhibition of pathological myocardial hypertrophy

Adiponectin deficiency exacerbates high-fat diet-induced myocardial hypertrophy and cardiac dysfunction, involving significant metabolic re-programming and oxidative stress. This is characterised by a return to a foetal metabolic state, namely, increased reliance on glycolysis and reduced efficiency of fatty acid oxidation. This metabolic reorganisation leads to cardiomyocyte hypertrophy and dysfunction. In this pathological process, adiponectin exerts a key protective role through multi-target intervention. By activating AMPK, adiponectin enhances fatty acid oxidation and glucose uptake in cardiomyocytes, thereby correcting metabolic disturbances, alleviating hypoxia, and inhibiting the drivers of pathological hypertrophy. Adiponectin inhibits the excessive production of reactive oxygen species (ROS), blocks ROS-mediated hypertrophy pathways such as angiotensin II (Ang II) and abnormal foetal gene expression, and promotes the production of the anti-inflammatory factor IL-10 by immune cells via AMPK, thereby preventing pathological hypertrophy. Its anti-inflammatory action further helps to maintain vascular endothelial function and myocardial perfusion by activating AMPK, thereby counteracting the Ang II-induced downregulation of the anti-hypertrophic microRNA miR-133a. Furthermore, it activates the PI3K/Akt survival pathway and acts synergistically with AMPK to attenuate Ang II-induced myocardial fibrosis and hypertrophy. This is crucial because chronic activation of the renin-angiotensin-aldosterone system (RAAS) promotes fibroblast activity, leading to excessive collagen deposition, myocardial stiffness and diastolic dysfunction (Sidhu et al., 2023). In addition to directly activating AMPK via AdipoR1/R2, adiponectin can also transmit signals via its binding protein, T-cadherin. T-cadherin regulates integrin levels and activates integrin-related kinase (ILK), thereby initiating the AMPK signalling pathway and preventing left ventricular hypertrophy. Consequently, the T-cadherin–integrin-ILK-AMPK axis provides an important mechanism for the cardioprotective effects of adiponectin that is independent of AdipoR (Denzel et al., 2010). In summary, through a multidimensional mechanism involving the correction of metabolic disorders, the counteraction of oxidative stress and the regulation of key signalling pathways, adiponectin establishes its core network, thereby inhibiting pathological myocardial hypertrophy and delaying the progression of heart failure.

### 7.4 Inhibition of myocardial apoptosis and fibrosis

By activating multiple signalling pathways to modulate apoptosis and fibrosis, adiponectin acts as a key protector of cardiac structural and functional integrity. Under ischaemic conditions, cardiovascular cells induce apoptosis in mesenchymal stem cells via the mitochondrial pathway; however, spheric adiponectin bound to AdipoR1 counteracts this process by activating AMPK. The combined use of adiponectin and mesenchymal stem cells significantly inhibits cardiomyocyte apoptosis and promotes angiogenesis in the myocardium surrounding the infarct zone (Tian et al., 2016; Tian et al., 2022). Furthermore, spheromalin can inhibit hypoxia/reoxygenation-induced necrotic apoptosis and apoptosis in cardiomyocytes by alleviating oxidative stress and suppressing the activity of the p38MAPK/NF-kappaB signalling pathway (Zhu K. et al., 2021). Histological analysis of a myocardial infarction model indicates that reduced wall thickness in the infarct margin is a key feature of adverse ventricular remodelling and ventricular wall aneurysm formation. Compared with standard myocardial infarction, adiponectin expression is significantly reduced in the border zone of the ventricular aneurysm model, a region of fate plasticity analogous to the penumbra in cerebral infarction. Adiponectin actively mediates crosstalk between adipocytes and macrophages following infarction and is crucial for regulating local inflammation and repair. Consequently, interventions targeting the border zone, particularly those aimed at rescuing this cardiac penumbra by increasing local adiponectin levels, represent a potential strategy for preventing ventricular aneurysm formation and the progression of myocardial infarction, which holds significant therapeutic implications for patients with coronary heart disease, obesity and/or type 2 diabetes (Jang et al., 2022). The anti-inflammatory effects of adiponectin are not limited to systemic regulation; they also play a crucial supporting role in local cardiac repair. It is worth noting that M2 macrophages play an important role in cardiac repair following myocardial infarction (Gianopoulos et al., 2025). The IL-6/adiponectin/HMGB1 feedback loop elucidates how the EAT, as a dynamic paracrine organ, participates in cardiac repair under stress conditions through interactions between immune and adipose cells. Adiponectin counteracts the progression of fibrosis and heart failure by directly inhibiting the excessive activation of fibroblasts; its protective effect is primarily achieved through the activation of the adiponectin-AMPK-PPARα axis, thereby alleviating the oxidative stress that drives fibrosis (Fujita et al., 2008).

### 7.5 Adiponectin and arrhythmias

Obesity may lead to uneven atrial and ventricular conduction via metabolic syndrome, haemodynamic changes (increased blood volume, ventricular diastolic dysfunction) and enhanced neurohormonal activity, ultimately increasing the risk of atrial fibrillation (AF), which is primarily manifested as P-wave prolongation (Meulendijks et al., 2024; Russo et al., 2008). Low levels of adiponectin are associated with the onset and progression of atrial fibrillation (Chen et al., 2024; Rafaqat et al., 2023). Although some studies have shown that higher circulating levels of adiponectin are associated with an increased risk of atrial fibrillation, this association may reflect a compensatory increase produced by the body to counteract inflammation and metabolic disturbances (Agbaedeng et al., 2022). The functional state of endothelial-like tissue (EAT) within adipose tissue plays a significant role in the pathogenesis of atrial fibrillation. A decline in EAT function, accompanied by adipocyte hypertrophy, is associated with a pro-inflammatory state, prolonged P-wave duration and reduced adiponectin levels, and may promote re-entry arrhythmias. It is worth noting that local adiponectin secretion levels in the EAT are a better predictor of atrial fibrillation risk than total circulating adiponectin levels. For example, in patients with post-operative atrial fibrillation, serum total adiponectin levels remained unchanged, whilst adiponectin levels in the EAT were significantly reduced. This suggests that even when systemic adiponectin levels are normal, a local deficiency of adiponectin in the EAT may still lead to cardiac adiponectin deficiency and play a key role in the pathogenesis of atrial fibrillation. There are significant gender differences in the association between adiponectin and atrial fibrillation. Adiponectin predicts the risk of atrial fibrillation only in women, acting as a positive regulator and longitudinal predictor of parasympathetic activity in women (Zhu T. et al., 2021). This suggests that adiponectin protects women from arrhythmias by stabilising heart rate and counteracting sympathetic overactivity through the enhancement of parasympathetic tone. These gender differences in the cardioprotective effects of adiponectin suggest that women may derive greater benefits from adiponectin in terms of autonomic nervous system regulation than men. Consequently, restoring and preserving the function of adiponectin particularly its local EAT function, rather than simply interpreting its circulating levels, is key to the prevention and treatment of atrial fibrillation, particularly in women.

Ventricular arrhythmias are one of the leading causes of sudden cardiac death following myocardial infarction, and their occurrence is primarily driven by excessive activation of the sympathetic nervous system. Adiponectin counteracts this process by modulating autonomic nervous function: it reduces central sympathetic tone and directly inhibits neural activity in the left stellate ganglion, a key sympathetic ganglion. This helps to stabilise the electrophysiological state of the ventricles and improve cardiac function following myocardial infarction. Notably, exogenous pre-treatment of the left stellate ganglion with adiponectin effectively prevents acute ischaemia-induced ventricular arrhythmias. The sharp decline in serum adiponectin levels within 72 h of acute myocardial infarction further highlights its crucial role in the prevention of arrhythmias (Zhou et al., 2022).

## 8 The adiponectin paradox

Although adiponectin has cardioprotective effects, epidemiological studies have found that, in elderly patients with cardiometabolic diseases or heart failure, higher adiponectin levels are associated with higher mortality rates; this phenomenon is known as the adiponectin paradox. Firstly, adiponectin levels are determined by the balance between its production and clearance; in the context of cardiovascular dysfunction, elevated levels may reflect a reduction in hepatic clearance rather than increased secretion. Secondly, high adiponectin levels may represent a compensatory response aimed at restoring cardiovascular homeostasis (Gianopoulos and Daskalopoulou, 2025). In individuals with elevated NT-proBNP levels, a significant dose-response relationship exists between adiponectin and cardiovascular mortality as well as all-cause mortality. This suggests that, when NT-proBNP is elevated, increased adiponectin levels may serve as a surrogate marker for an increased risk of mortality (Mukama et al., 2023). For every two-fold increase in NT-proBNP, adiponectin concentration rises by 11.4 per cent; NT-proBNP levels influence adiponectin levels, whilst there is no correlation between adiponectin and cardiac function parameters (Christen et al., 2021). Individuals with higher adiponectin levels exhibit better cardiovascular and metabolic parameters than those with lower adiponectin levels (Abushamat et al., 2025). Furthermore, this paradox manifests differently across various types of heart failure. In heart failure with reduced ejection fraction (HFrEF), stretching of cardiac myocytes stimulates the release of NP, thereby promoting adiponectin secretion and potentially leading to adiponectin resistance. By contrast, more than half of patients with HFpEF are obese; their pathological changes are primarily characterised by inflammation and increased myocardial stiffness, and are not usually accompanied by significant NP release. This distinction sheds light on the relationship between adiponectin and mortality from a subtype-specific perspective.

HMW is the most bioactive form of adiponectin; the biphasic nature of adiponectin is primarily attributable to differences in the HMW fraction within the molecule, and the ratio of HMW to total adiponectin (HMWR) has greater predictive value than total adiponectin alone. In patients with chronic heart failure, higher total APN levels are associated with an increased mortality rate; however, HMWR levels are of limited value in predicting mortality. In contrast, for patients with acute heart failure, higher HMWR levels and their changes may play a primary role in cardioprotection and serve as a better prognostic indicator than chronic heart failure (Xu et al., 2005). The obesity paradox exists in cardiovascular disease, whereby a higher BMI is associated with a reduced incidence of atrial fibrillation and heart failure. Some researchers attribute this to inappropriate nutritional indicators, pointing out that the paradox disappears once confounding factors are adjusted for. Other researchers, however, have reported that even after adjusting for confounding factors, obesity remains associated with fewer adverse events; the energy reserves provided by obesity may exert a protective effect during high-energy-demand states such as heart failure. This view is supported by findings that mice fed moderate- and high-fat diets exhibited larger vessel diameters, thicker endothelial glycocalyx and greater stress tolerance (Zhang Z. Z. et al., 2025). In summary, the adiponectin paradox highlights the complex relationship between biomarker levels and clinical outcomes. Adiponectin is not an independent risk factor; it must be considered in conjunction with cardiac function, heart failure phenotype, metabolic status and clearance capacity. It is therefore inappropriate to categorise it simplistically as either protective or harmful. In future, a nuanced and context-specific approach will be required to interpret its role. Furthermore, its value as a cardiac risk marker needs to be validated by comparison with established indicators such as blood lipids, blood glucose and homocysteine.

## 9 Prospects for adiponectin-based therapies

Various weight management interventions can modulate adiponectin levels. In addition to calorie restriction, the effects of which vary according to gender and the menstrual cycle, for example, the impact on premenopausal women is negligible, aerobic exercise, bariatric surgery and GLP-1 receptor agonists can all increase circulating adiponectin levels. Mechanistically, sustained calorie restriction improves adipose tissue plasticity and remodels the extracellular matrix. However, it cannot fully reverse obesity-induced adipose tissue dysfunction, and long-term clinical data are lacking. Certain drugs and nutrients such as statins, thiazolidinediones and vitamins can also upregulate adiponectin levels. It is worth noting that certain interventions exhibit gender differences: rosiglitazone promotes the browning of white adipose tissue and activates PPARγ, thereby improving lipid metabolism and energy balance, and exhibits a stronger insulin-sensitising effect in male models. Exercise, metformin and bariatric surgery can reduce ceramide levels in muscle. Overall, it remains unclear whether gender differences exist in most obesity treatments and their cardiovascular benefits, and further research is required.

## 10 Conclusion

In the context of the obesity epidemic, adiponectin has been shown to be a key regulator of cardiovascular health, exhibiting sex-specific differences in its action. Its wide-ranging cardioprotective functions including the regulation of myocardial energy metabolism, inflammation, oxidative stress and fibrosis are fundamentally influenced by sex-specific differences in fat distribution, hormonal regulation and mitochondrial function. Recognising these gender differences is crucial for moving beyond a one-size-fits-all treatment approach and provides a strong basis for developing gender-specific strategies to improve the prevention and management of obesity-related cardiac complications.
